# Megaprosthetic reconstruction of the distal femur with a short residual proximal femur following bone tumor resection: a systematic review

**DOI:** 10.1186/s13018-023-03553-7

**Published:** 2023-01-27

**Authors:** Shinji Tsukamoto, Andreas F. Mavrogenis, Tomoya Masunaga, Akira Kido, Kanya Honoki, Yuu Tanaka, Hiromasa Fujii, Yasuhito Tanaka, Costantino Errani

**Affiliations:** 1 Department of Orthopaedic Surgery, Nara Medical University, 840, Shijo-Cho, Kashihara-City, Nara 634-8521 Japan; 2 First Department of Orthopaedics, School of Medicine, National and Kapodistrian University of Athens, 41 Ventouri Street, 15562 Holargos, Athens, Greece; 3 Department of Rehabilitation Medicine, Nara Medical University, 840, Shijo-Cho, Kashihara-City, Nara 634-8521 Japan; 4 Department of Rehabilitation Medicine, Wakayama Professional University of Rehabilitation, 3-1, Minamoto-Cho, Wakayama-City, Wakayama 640-8222 Japan; 5 Department of Orthopaedic Oncology, IRCCS Istituto Ortopedico Rizzoli, Via Pupilli 1, 40136 Bologna, Italy

**Keywords:** Distal femur replacement, Distal femoral tumor, Short proximal femur, Compressive osseointegration, Allograft prosthetic composite, Custom-made megaprosthesis

## Abstract

**Background:**

To investigate the risk of postoperative function and complications associated with reconstruction methods in patients with short residual proximal femurs (< 12 cm) after resection of distal femoral bone tumors, we performed a systematic review of studies reporting postoperative function and complications in these patients.

**Methods:**

Of the 236 studies identified by systematic searches using the Medline, Embase, and Cochrane Central Register of Controlled Trials databases, eight were included (none were randomized controlled trials). In these studies, 106 (68.4%), 12 (7.7%), and 37 (23.9%) patients underwent reconstruction with custom-made megaprostheses with extracortical plates or cross-pins, allograft prosthetic composite (APC), and Compress^®^ compliant pre-stress (CPS) implants, respectively.

**Results:**

Aseptic loosening occurred slightly more frequently in the APC group than in the other reconstruction methods (APC group, 21%; custom-made megaprosthesis group, 0–17%; CPS implant group, 14%). No differences were noted in the frequencies of implant breakage, fractures, or infections between the three reconstruction methods. Mechanical survival, where endpoint was set as implant removal for any reason, was 80% at seven years in the APC group, 70–77% at 10 years in the custom-made megaprosthesis group, and 68% at nine years in the CPS implant group. Therefore, there appeared to be no difference among the three reconstruction methods with respect to mechanical survival.

**Conclusions:**

During megaprosthetic reconstruction of the distal femur with a short residual proximal femur after bone tumor resection, similar results were obtained using custom-made megaprostheses, APCs, and CPS implants.

**Supplementary Information:**

The online version contains supplementary material available at 10.1186/s13018-023-03553-7.

## Background

Primary malignant bone tumors, such as osteosarcoma and Ewing's sarcoma, often arise in the distal femur [1]. Reconstruction of the distal femur after tumor resection is challenging in young patients with long-life expectancy and high functional demands [2]. Reconstruction with a megaprosthesis allows early weight bearing, relatively short hospitalization, early return to daily activities, and early resumption of postoperative chemotherapy [3, 4]. Occasionally, tumors extend to the subtrochanteric area, requiring subtrochanteric femoral osteotomy during tumor resection, and the remaining proximal femur becomes too short to allow insertion of a standard modular-type megaprosthesis stem (120–150 mm) [5, 6]. Even with short-stem prostheses, aseptic loosening occurs at a high rate because of the limited stem-to-bone interface [7]. When it is no longer possible to preserve the remaining proximal femur, total femur replacement can be performed, but this leads to a significant loss of function compared to distal femoral replacement [8–10]. Additionally, total femur replacement is associated with a risk of dislocation and impaired hip abduction function [11]. Therefore, to preserve hip abduction function, it is important to preserve the remaining short proximal femur [11].

**Fig. 1:**
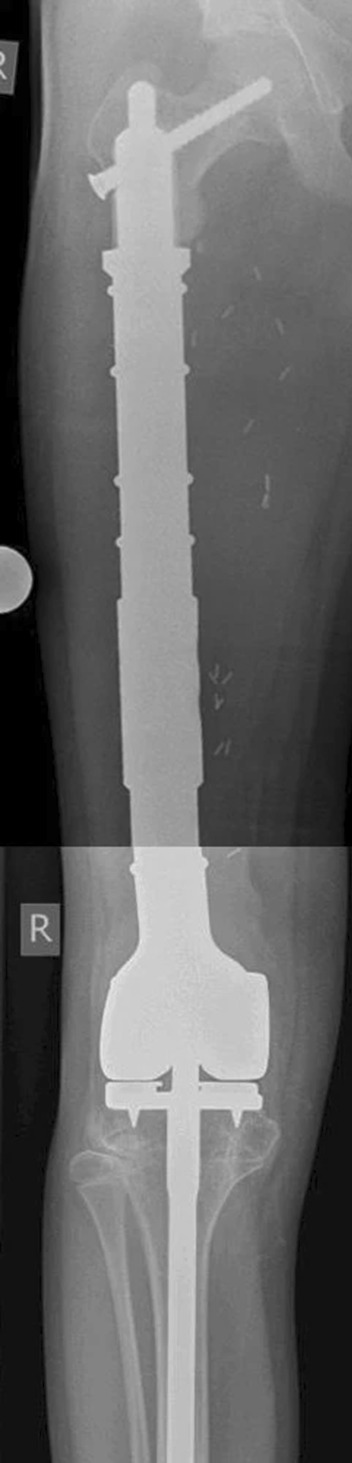
Non-cemented, custom-made short-stem megaprosthesis with cross-pins (cited from Dieckman et al. [13])

**Fig. 2:**
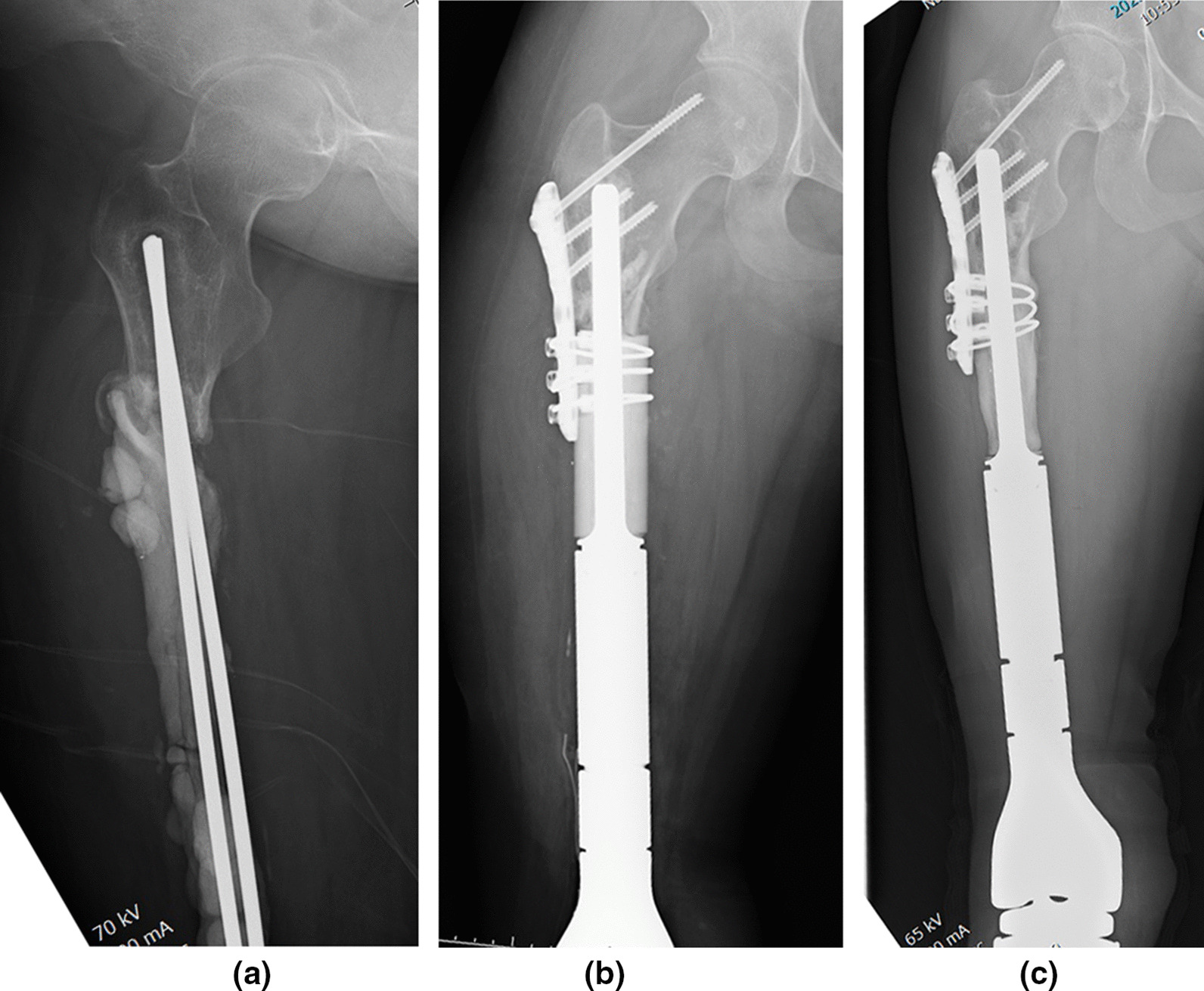
**a** Infection in an 18-year-old female after resection of the distal femoral osteosarcoma and reconstruction with a megaprosthesis. After removal of the prosthesis and debridement, antibiotic cementing and knee arthrodesis were performed to cure the infection, but the remaining proximal femur was short. **b** X-ray immediately after reconstruction of the allograft prosthetic composite. Cement fixation and additional plate fixation were also performed. **c** Three years post-operatively, the host bone and allograft fused, and no aseptic loosening was observed

Custom-made megaprostheses with extra-cortical plates or cross-pins (Fig. 1) [5, 11–15], allograft prosthetic composite (APC) implants (Fig. 2) [2], and Compress^®^-compliant pre-stress (CPS) implants [16] have been used as reconstructive options for patients with a short proximal femur remaining after resection of a distal femoral bone tumor. However, the results of each reconstruction method are limited to retrospective case series, owing to their rarity, and there are no comparative studies or randomized controlled trials (RCTs). Therefore, the optimal reconstruction method remains unknown. To the best of our knowledge, no systematic review of the literature has addressed this issue.

To investigate the risk of postoperative function and complications in patients with a short residual proximal femur after distal femoral bone tumor resection, we performed a systematic review of studies reporting the functional results and complications in these patients after reconstruction with custom-made megaprostheses, APC, and CPS implants.

## Methods

We followed the recommendations of the Preferred Reporting Items for Systematic Reviews and Meta-analyses 2020 statement [17]. Additionally, we registered our protocol with the University Hospital Medical Information Network Clinical Trials Registration as UMIN000048111 (http://www.umin.ac.jp/ctr/index.htm [accessed on June 20, 2022]).

### Eligibility criteria

Only studies that reported functional results and complications of megaprosthetic reconstruction of the distal femur with a short residual proximal femur following bone tumor resection were included. A residual short proximal femur was defined as < 12 cm in length, where the standard modular-type megaprosthesis stem could not be inserted. Studies in which reconstruction was performed in patients without short residual proximal femurs were excluded. Studies that performed reconstruction in patients with short residual proximal femurs, but did not specify both functional results and complications, were also excluded. Only English- and Japanese-language literatures were included, with no restrictions on publication year.

### Literature search and study selection

The literature was searched according to a systematic search strategy using the Medline, Embase, and Cochrane Central Register of Controlled Trials databases on May 31, 2022 (Additional file 1). Additionally, the bibliographies of the retrieved literature were used to identify other relevant studies.

### Data collection and presentation

The studies were independently selected, and data were extracted. In case of disagreement, an agreement was reached among the authors. The following data were collected using a data collection sheet: (1) Basic data (author name, year of publication, journal name, type of study, period investigated, number of patients, patient age, and tumor histology); (2) Surgical indication, method of reconstruction, cement fixation, porous coating, time to full weight bearing, stem length of prosthesis, adjuvant chemotherapy, adjuvant radiotherapy, non-union, aseptic loosening, implant breakage, fracture, infection, mechanical survival (where the endpoint was set to implant removal for any reason), oncological outcome, Musculoskeletal Tumor Society (MSTS) score [18], and postoperative follow-up period.

### Data summary

Tables 1 and 2 summarize the data extracted from the collected data. Table 3 summarizes the time to full weight-bearing, non-union, aseptic loosening, implant breakage, fracture, infection, mechanical survival, MSTS score [18], and postoperative follow-up period for each reconstruction method (custom-made megaprosthesis, APC, and CPS implant). All studies included in this review were non-randomized; therefore, data pooling (meta-analysis) was not appropriate and thus, not performed.

**Table 1:** Overall study characteristics

Author	Year	Type of study	Period investigated	Number of patients	Age (years)	Histology	Indication	Type of reconstruction	Cement fixation	Porous coating
Christ et al. [11]	2021	SR	2002 to 2018	14	Mean: 36	Osteosarcoma (10), Ewing sarcoma (2), Pleomorphic sarcoma (1), and GCTB (1)	The remaining short proximal femur was used in 24%, and 8/14 retained ≤ 20% of their femur	Short-stemmed endoprostheses with custom cross-pin and extra-cortical plates fixation	None	Yes
Hindiskere et al. [2]	2021	SR	2008 to 2018	14	Median: 14 (range, 7–18)	Osteosarcoma (14)	Length of proximal femur remaining after tumor resection was < 12 cm	Allograft prosthetic composite	None: 5, Only in allograft: 3, In allograft and host bone: 6	NA
Bernthal et al. [12]	2019	SR	1980 to 2017	36	Mean: 33	Osteosarcoma (5), Chondrosarcoma (4)	(1) Projected stem length of ≤ 9 cm; (2) < 2 cm of residual diaphyseal bone; or (3) early (within two years) aseptic loosening of a long stem due to rotational instability	Short-stemmed endoprostheses with custom cross-pin fixation	All	No
Stevenson et al. [5]	2017	SR	1998 to 2013	13	Mean: 26	NR	A short stem was defined as < 100 mm	Short-stemmed endoprostheses with extra-cortical plates	None	Yes
Calvert et al. [16]	2014	MR	NR	37	Mean: 21	Osteosarcoma (47)	Patients with a short proximal femur remaining after tumor resection and unable to insert a standard-length stem	Compressive osseointegration	None	Yes
Dieckmann et al. [13]	2014	SR	2003 to 2012	15	Mean: 33 (range, 11–73)	Osteosarcoma (9), Ewing sarcoma (4), Chondrosarcoma (2), and Myxofibrosarcoma (1)	Remaining proximal femur after tumor resection should be at least 40 mm	Short-stemmed endoprostheses with custom cross-pin fixation	None	Yes
Cobb et al. [14]	2005	SR	1987 to 1988	6	Mean: 32 (range, 9–72)	Osteosarcoma (4), Ewing sarcoma (1), and GCTB (1)	The remaining distal end of the femur after tumor resection was within 50 mm of the lesser trochanter	Triplate fixation	None	Yes
Cannon et al. [15]	2003	SR	1988 to 2001	22	Range: 6–68	Osteosarcoma (6), Ewing sarcoma (3), Chondrosarcoma (2), and MFH (1)	Patients with short proximal femur remaining after tumor resection	Short-stemmed endoprostheses with custom cross-pin fixation	All	No

*SR* single institutional non-randomized retrospective study; *MR* multi-institutional non-randomized retrospective study; *GCTB* giant cell bone tumor; *MFH* malignant fibrous histiocytoma; *NA* not applicable; *NR* not reported

**Table 2:** Overall study characteristics

Author	Time to full weight bearing (months)	Length of endoprosthetic stem (mm)	Adjuvant chemotherapy	Adjuvant radiotherapy	Non-union, aseptic loosening	Implant breakage	Fracture	Infection	Mechanical survival	Oncological outcome	MSTS score	Follow-up (months)
Christ et al. [11]	NR	68	NR	NR	Aseptic loosening (14%)	21% (screw breakage: 7%, fractures of the taper at the modular junction between the custom stem and the rest of the modular prosthesis: 14%)	0	7%	NR	Local recurrence (0)	Mean: 24 (range, 16–30)	Mean: 72
Hindiskere et al. [2]	Median: 7 (range, 6–9)	Median: 135 (range, 120–165)	29%	NR	Non-union (0), aseptic loosening (21%)	7%	0	7%	Mechanical survival at 7 years: 80%	CDF (13) DOD (1)	Median: 27 (range, 22–30)	Median: 49
Bernthal et al. [12]	NR	NR	14%	NR	Aseptic loosening (8%)	11% (a fatigue fracture of the stem through cross-pin holes)	0	17%	Mechanical survival at 5 years: 82%, 10 years: 77%, 15 years: 77%	Local recurrence (2)	NR	Median: 132
Stevenson et al. [5]	NR	Mean: 79 (range, 34–100)	NR	15%	Aseptic loosening (0)	8%	0	8%	Mechanical survival at 10 years: 70%	Local recurrence (0)	NR	Mean: 84 (range, 12–204)
Calvert et al. [16]	1.5 months non-weight bearing	NR	3%	0	Aseptic loosening (14%)	0	2%	12%	Mechanical survival at 2 years: 68%	NR	NR	Mean: 68 (range, 31–113)
Dieckmann et al. [13]	NR	Mean: 77 (range, 45–130)	NR	NR	Aseptic loosening (13%)	7% (screw breakage)	7%	7%	NR	CDF (8), AWD (2), DOD (5)	Mean: 23 (range, 9–28)	Mean: 47 (range, 5–95)
Cobb et al. [14]	1.5	Mean: 60 (range, 40–70) (Length of plate)	NR	NR	Aseptic loosening (17%)	0	0	0	NR	CDF (5), DOD (1)	Mean: 28 (range, 27–30)	Mean: 67 (range, 11–93)
Cannon et al. [15]	NR	NR	NR	NR	Aseptic loosening (5%)	5% (a fatigue fracture of a Morse taper trunnion of a custom-casted component)	0	0	NR	NR	NR	Median: 57 (range, 25–182)

*NR* not reported; *MSTS* Musculoskeletal Tumor Society; *CDF* continuous disease-free; *DOD* die of disease; *AWD* alive with disease

**Table 3:** Summary of complications and functional outcomes for each reconstruction method

Reconstruction	Time to full weight bearing (months)	Non-union	Aseptic loosening	Implant breakage	Fracture	Infection	Mechanical survival	MSTS score	Mean (median) follow-up period (months)
Custom-made megaprosthesis with extra-cortical plates or cross-pin (*n* = 106)	1.5	NA	0–17% (Cement fixation: 5–8%, Porous coating: 0–17%)	0–21%	0–7%	7–17%	70–77% at 10 years	23–28	47–132
Allograft prosthetic composite (*n* = 14)	7	0%	21%	7%	0%	7%	80% at 7 years	27	49
Compress^®^ Compliant Pre-stress implant (*n* = 37)	1.5	NA	14%	0%	2%	12%	68% at 9 years	NR	68

*MSTS* Musculoskeletal Tumor Society; *NA* not applicable; *NR* not reported

### Assessment of methodological quality

The methodological quality of each study was independently assessed. When there was disagreement, agreement was reached among the authors through discussion. Articles included in the final analysis were independently assessed according to the Risk of Bias Assessment tool for Non-randomized Studies (RoBANS tool) to assess the quality of non-randomized studies in meta-studies [19].

### Search results

**Fig. 3:**
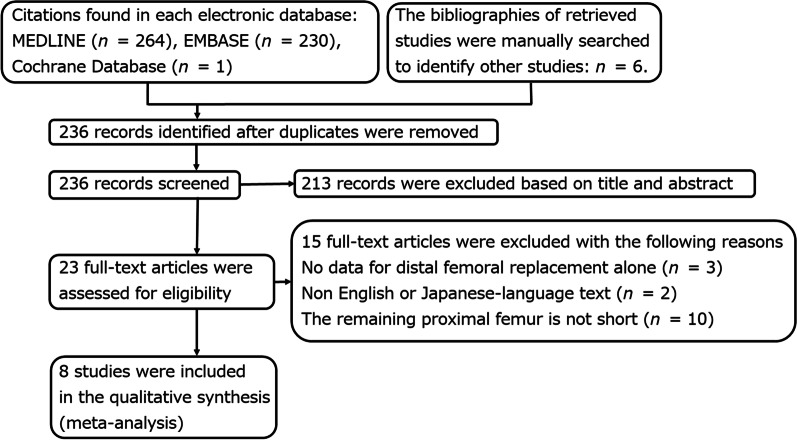
Flow chart of the search for relevant articles

Of the 236 studies identified by the search, eight were included in our review (Fig. 3; Tables 1, 2 and 3) [2, 5, 11–16]. These eight studies were not RCTs.

### Demographic data and ratio of patients who underwent reconstruction with custom-made megaprostheses, APC, and compression-compliant pre-stress implant

A total of 155 patients underwent megaprosthetic reconstruction of their distal femurs with a short residual proximal femur, following bone tumor resection. Of the 155 patients, 106 (68.4%) underwent reconstruction with a custom-made megaprosthesis, 12 (7.7%) with APC, and 37 (23.9%) with CPS implants (Tables 1, 2 and 3). Custom-made megaprostheses were the most commonly used from the 1980s until recently (Table 1). Between 2008 and 2018, at one institution, APC was the only method used to reconstruct the remaining short proximal femur after bone tumor resection of the distal femur [2] (Table 1).

### Methodological quality of the included studies

The quality of each study was assessed using the RoBANS tool; overall, there was a moderate risk of bias. All the eight included studies showed that ‘selection of participants’ and ‘confounding variables’ were high, while ‘measurement of exposure,’ ‘blinding of outcome,’ ‘incomplete outcome data,’ and ‘selective outcome reporting’ were low.

## Results

Time to full weight bearing was 1.5 months in the group reconstructed with custom-made megaprostheses or CPS implants [14, 16] compared with seven months in the group reconstructed with APCs [2]. The time to full weight bearing was longer in the APC group due to the need for non-weight bearing until the allograft fused with the remaining host bone [2].

The frequency of aseptic loosening was 21% in the group reconstructed with APCs [2], 0–17% in the group reconstructed with a custom-made megaprosthesis [5, 11–15], and 14% in the group reconstructed with CPS implants [16]. This indicates that the APC group may have a slightly higher frequency of loosening than the other reconstruction methods; however, the quality of these studies was not good, and they were not RCTs. Therefore, we could not pool the data and perform statistical analyses to determine whether there were differences in the occurrence of aseptic loosening among the three treatment options. For custom-made megaprostheses, the frequency of aseptic loosening ranges between 0 and 17% in the porous coating group [5, 11, 13, 14] and 5 and 8% in the cement fixation group [12, 15]. There was no difference in the frequency of aseptic loosening between the porous coating and cement fixation groups.

The frequencies of implant breakage were 0–21% [5, 11–15], 7% [2], and 0% [16] in the custom-made megaprosthesis, APC, and CPS implant groups, respectively. The breakdown of implant breakage was 7% for screw breakage [11, 13], 5–14% for component connection breakage [11, 15], and 11% for stem breakage [12]. Additionally, the frequency of fractures was 0–7% [5, 11–15], 0% [2], and 2% [16] in the custom-made megaprosthesis, APC, and CPS implant groups, respectively. Moreover, the frequency of infection was 7–17% [5, 11–15], 7% [2], and 12% [16] in the custom-made megaprosthesis, APC, and CPS implant groups, respectively. Therefore, the frequencies of implant breakage, fracture, and infection are similar among the three reconstruction methods [2, 5, 11–16, 20].

Mechanical survival, where the endpoint was set for implant removal for any reason, was 70–77% at 10 years in the group reconstructed with a custom-made megaprosthesis [5, 12], 80% at 7 years in the group reconstructed with APCs [2], and 68% at 9 years in the group reconstructed with a CPS implant [16]. Therefore, there appears to be no difference between the three reconstruction methods with respect to mechanical survival [2, 5, 12, 16].

## Discussion

The most appropriate reconstructive approach for patients with a short residual proximal femur after distal femoral bone tumor resection remains unclear. We included studies that reported megaprosthetic reconstruction of the distal femur with a short residual proximal femur after bone tumor resection and compared the mechanical survival and risk of complications between custom-made megaprostheses, APC, and CPS implants. The APC group had a longer time to full weight bearing because of the need for non-weight bearing until the allograft was fused with the remaining host bone. The APC group also appeared to have a slightly higher frequency of aseptic loosening than the other reconstruction methods groups. There was no difference in the frequency of aseptic loosening between the porous coating and cement fixation groups for the custom-made megaprostheses. Additionally, there were no differences in the frequencies of implant breakage, fracture, or infection between the three reconstruction methods. There appeared to be no difference among the three reconstruction methods with respect to mechanical survival. Therefore, all the custom-made prostheses, APC, and CPS implants may be good choices for reconstruction of the distal femur in patients with a short residual proximal femur after bone tumor resection.

This study has several limitations. First, all studies included in this systematic review were retrospective and bias based on patient background. All studies in which surgical indications were made for patients who did not have a short residual proximal femur were excluded; however, there were still variations in surgical indications. However, RCTs can avoid many of these biases by randomly allocating participants into groups. As we identified no RCTs, further well-designed cohort and observational studies with strong effects may provide more reliable information. Second, 14 patients underwent APC reconstruction of the distal femur with short residual proximal femurs after resection of the bone tumors, and 37 patients underwent CPS implant reconstruction. The number of patients who underwent these reconstruction procedures is small. Future studies with larger populations and longer follow-up periods may yield different results. However, to date, this is the extent of information available regarding reconstruction of the distal femur in patients with short residual proximal femurs after bone tumor resection. Third, the time span of the cases included in the study is large; from 1980 to 2021, the time span is as long as 40 years. Too long a time span changes the understanding of the surgeon, the design of the prosthesis, the materials, and processes for making the prosthesis. Even in the same center, it is difficult to achieve the same clinical solutions for similar cases. However, because of the rarity of patients requiring residual proximal femoral reconstruction after distal femoral bone tumor resection, we did not limit the year of publication to ensure a sufficient number of cases.

APC reconstruction combines a modular megaprosthesis with a massive bone allograft [21]. Cement is often required for proper stem fixation of massive bone allografts because avascular allografts do not lead to bone growth into the porous coated stem, similar to the host bone [2]. The use of plates or screws to increase allograft fixation to the host bone has also been recommended [2]. Healey et al. proposed telescopic allograft reconstruction, in which the allograft and host bone overlap like a telescope to promote allograft–host bone union [22]. Healey et al. recommended an overlap length of 50 mm [22]. In their study, Hindiskere et al. reported that all 14 patients achieved allograft–host bone union with an average overlap length of 19 mm [2]. In this systematic review, the frequency of loosening was slightly higher in the APC reconstruction group than that in the other reconstruction groups. Allograft resorption may be a cause of loosening, as bone union was observed in all cases [2]. The advantage of using APC is that the procedure can be customized based on the surgeon’s needs at the time of surgery by combining a modular megaprosthesis with an allograft [20, 21]. Additionally, during reconstruction at the time of the first surgery, it is possible to use a recycled autograft instead of an allograft to reconstruct the residual short proximal femur in combination with a modular-type megaprosthesis [7].

Custom-made megaprostheses comprise a short stem with extracortical plates or cross-pins that are cemented or fixed with a porous coating to induce osteointegration. However, they are time-consuming to manufacture and can be problematic in patients who do not require neoadjuvant chemotherapy [23]. Based on the results of our systematic review, there was no difference in the frequency of aseptic loosening between cemented and porous coating fixation of custom-made megaprostheses. In a previous study, Hu et al. treated 85 patients with custom-made cemented or cementless fixed total knee systems (United USTAR system) with a follow-up period of 89 months [24]. At five years, the mechanical survival rate was 75% in the cemented group and 94% in the cementless group [24]. Causes of failure included aseptic loosening in five patients and implant breakage in six patients in the cemented stem group [24]. Mechanical survival in the cementless fixation group was significantly better than that in the cemented group (p = 0.01) [24].

CPS implants (Biomet, Warsaw, IN, USA) use the principles of Wolff's law to generate compliant self-adjusting compression via a short (4 or 8 cm) intramedullary traction bow that produces a compressive force, which promotes biological fixation [25, 26]. The CPS implant was approved by the Food and Drug Administration in 2003 because it demonstrated complication rates and functional outcomes comparable to those of cemented stems (Orthopaedic Salvage System [OSSTM]; Biomet) [27]. CPS implants have been shown to induce bone hypertrophy at the distal bone–prosthetic junction of a stable and fixed implant [28, 29]. The insertion of a relatively less rigid medullary fixation component eliminates the need for stress shielding that occurs with conventional stem devices, as stresses are transferred directly to the bone–prosthetic interface during normal cyclic loading [26, 28]. These features make this implant an attractive option for reconstruction of the remaining short proximal femur. However, the indications for CPS implants are narrower than those for APCs and custom-made megaprostheses, as the indications are limited to the remaining proximal femur with a minimum cortical thickness of 2.5 mm, without cortical defects or osteolysis [6, 27]. In the case of revision in previously reconstructed patients, the cortical thickness may not be sufficient to achieve compression of the CPS implant [27].

The goal of megaprosthetic reconstruction of the distal femur in patients with short residual proximal femurs is to preserve the hip and abductor muscle mechanism. Loss of abductor muscle strength increases the energy expenditure of gait by 1.41 times the normal [30], and patients who undergo proximal femoral replacement experience significantly lower functional outcomes [8–10]. Kalra et al. reported a mean MSTS score of 72% in 26 patients who underwent total femur replacement and were followed for an average of 57 months [10]. Furthermore, other authors have reported similar MSTS scores of 60–70% [8, 9]. Therefore, it is essential to maintain hip and abductor attachments, especially in young patients with long life expectancy to preserve function and reduce the risk of complications and revision surgeries [11].

## Conclusions

**Fig. 4:**
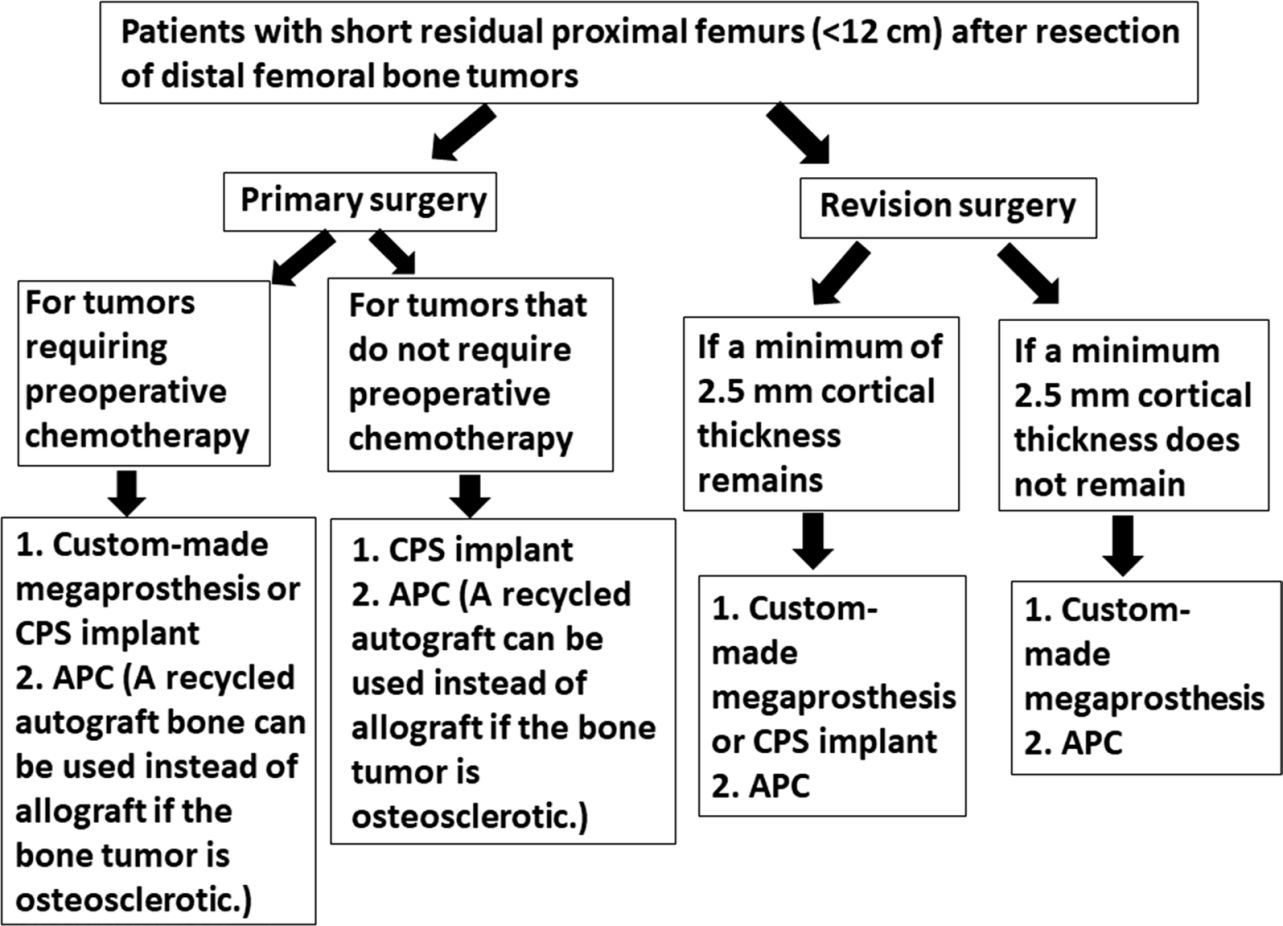
Algorithm for megaprosthetic reconstruction of the distal femur with a short residual proximal femur following bone tumor resection (CPS: Compress^®^ compliant pre-stress; APC: allograft prosthetic composite)

In megaprosthetic reconstruction of the distal femur in patients with a short residual proximal femur after bone tumor resection, the time to full weight bearing was longer in the APC group. Aseptic loosening was slightly more frequent in the APC group (21%) than in other reconstruction methods (0–17%). There were no differences in the incidence of implant breakage, fracture, or infection between the three reconstruction methods. Regarding mechanical survival, there was no difference between the three reconstruction methods. Indications for CPS implants are limited to those with a minimum remaining cortical thickness of 2.5 mm without cortical loss or osteolysis in the remaining proximal femur. Therefore, reconstruction of the residual short proximal femur after bone tumor resection of the distal femur had similar results with the CPS implants, APCs, and custom-made tumor prostheses; reconstruction with APC or a custom-made prosthesis is preferable in patients with thinning of the bone cortex of the residual short proximal femur. We propose an algorithm for megaprosthetic reconstruction of the distal femur with a short residual proximal femur following bone tumor resection (Fig. 4). However, only a small number of patients were included in this systematic review, and only retrospective studies were available. To revalidate our results, more detailed patient reports (allowing patients to be pooled for subsequent analysis) and obligatory follow-up of tumor reconstructions with some sort of registry may be required.

## Supplementary Information

**Additional file1.** Search strategy.

## Data Availability

All data generated or analyzed during this study are included in this published article.

## Abbreviations

APC: Allograft prosthetic composite
CPS: Compress^®^ Compliant Pre-stress
RCT: Randomized controlled trial
MSTS: Musculoskeletal tumor society
RoBANS: Risk of Bias Assessment tool for Non-randomized Studies
