# Supplementary material for: Megaprosthetic reconstruction of the distal femur with a short residual proximal femur following bone tumor resection: a systematic review
Source: J Orthop Surg Res. 2023 Jan 27;18:68. doi: 10.1186/s13018-023-03553-7 (PMC9881341; doi:10.1186/s13018-023-03553-7)
Supplement: Supplementary file 1 — Additional file1. Search strategy. [file 13018_2023_3553_MOESM1_ESM.docx]

| Databases: MEDLINE® | | 2022/5/31 |
| --- | --- | --- |
| Set# | Searched for | Results |
| S1 | (MESH.EXACT.EXPLODE("Bone Neoplasms -- surgery")) OR (TI,AB((bone N/3 (tumo* OR sarcoma*)) OR osteosarcoma* OR oncologi*)) | 159416* |
| S2 | (MESH.EXACT.EXPLODE("Allografts" OR "Transplantation, Homologous" OR "Bone Transplantation" OR "Prosthesis Design" OR "Osseointegration" OR "Prosthesis Implantation" OR "Hip Prosthesis")) OR (TI,AB(allograft* OR custom* OR compress*)) | 639396* |
| S3 | (TI,AB(((short OR residual* OR remain* OR reconstruct*) N/3 proximal N/3 fem*) OR megaprosthe* OR (massive N/2 endoprosthe*) OR (large N/2 fem* N/2 defect*))) | 984° |
| S4 | (S1 and S2 and S3) | 264° |
|  |  |  |
| * Duplicates are removed from the search, but included in the result count. | |  |
| ° Duplicates are removed from the search and from the result count. | |  |
|  |  |  |
| Databases: Embase® | | 2022/5/31 |
|  | an(52481731 OR 2016966977 OR 634412850) |  |
| Set# | Searched for | Results |
| S1 | (EMB.EXACT.EXPLODE("bone tumor -- surgery")) OR (TI,AB((bone N/3 (tumo* OR sarcoma*)) OR osteosarcoma* OR oncologi*)) | 226880* |
| S2 | (EMB.EXACT.EXPLODE("bone transplantation" OR "allograft" OR "allotransplantation" OR "prosthesis design" OR "osseointegration" OR "prosthesis implantation" OR "hip prosthesis")) OR (TI,AB(allograft* OR custom* OR compress*)) | 624528* |
| S3 | (TI,AB(((short OR residual* OR remain* OR reconstruct*) N/3 proximal N/3 fem*) OR megaprosthe* OR (massive N/2 endoprosthe*) OR (large N/2 fem* N/2 defect*))) | 1140° |
| S4 | (S1 and S2 and S3) | 230° |
|  |  |  |
| * Duplicates are removed from the search, but included in the result count. | |  |
| ° Duplicates are removed from the search and from the result count. | |  |
|  |  |  |
| Cochrane |  | 2022/5/31 |
| ID | Search | Hits |
| #1 | [mh "Bone Neoplasms"/SU] OR (((bone NEAR/2 tumo*) OR (bone NEAR/2 sarcoma*) OR osteosarcoma* OR oncologi*):ti,ab,kw) | 7479 |
| #2 | ([mh "Allografts"] OR [mh "Transplantation, Homologous"] OR [mh "Bone Transplantation"] OR [mh "Prosthesis Design"] OR [mh "Osseointegration"] OR [mh "Prosthesis Implantation"] OR [mh "Hip Prosthesis"]) OR ((allograft* OR custom* OR compress*):ti,ab,kw) | 34887 |
| #3 | ((short NEAR/3 proximal NEAR/3 fem*) OR (residual* NEAR/3 proximal NEAR/3 fem*) OR (remain* NEAR/3 proximal NEAR/3 fem*) OR (reconstruct* NEAR/3 proximal NEAR/3 fem*) OR megaprosthe* OR (massive NEAR/2 endoprosthe*) OR (large NEAR/2 fem* NEAR/2 defect*)):ti,ab,kw | 21 |
| #4 | #1 and #2 and #3 | 1 |
